# Using data from patient interactions in primary care for population level chronic disease surveillance: The Sentinel Practices Data Sourcing (SPDS) project

**DOI:** 10.1186/1471-2458-14-557

**Published:** 2014-06-05

**Authors:** Abhijeet Ghosh, Karen E Charlton, Lisa Girdo, Marijka Batterham

**Affiliations:** 1 Grand Pacific Health Ltd. trading as Illawarra-Shoalhaven Medicare Local (ISML), Wollongong, Australia; 2 School of Medicine, Faculty of Science, Medicine & Health, University of Wollongong (UOW), Wollongong, Australia; 3 Statistical Consulting Centre, National Institute of Applied Statistics Research Australia, University of Wollongong (UOW), Wollongong, Australia

**Keywords:** Sentinel sites, Surveillance, Primary care, General practice, Morbidity data

## Abstract

**Background:**

Population health planning within a health district requires current information on health profiles of the target population. Information obtained during primary care interactions may provide a valuable surveillance system for chronic disease burden. The Sentinel Practices Data Sourcing project aimed to establish a sentinel site surveillance system to obtain a region-specific estimate of the prevalence of chronic diseases and mental health disorders within the Illawarra-Shoalhaven region of New South Wales, Australia.

**Methods:**

In September 2013, de-identified information for all patient interactions within the preceding 24 months was extracted and collated using a computerised chronic disease management program that has been designed for desktop application (Pen Computer Systems Clinical Audit Tool: ^™^ (PCS CAT)). Collated patient data included information on all diagnosed pathologies and mental health indicators, clinical variables such as anthropometric measures, and patient demographic variables such as age, sex, geographical location of residence and indigenous status. Age-standardised prevalence of selected health conditions was calculated.

**Results:**

Of the 52 general practices within the 6 major Statistical Local Areas (SLAs) of the health district that met the inclusion criteria, 17 consented to participate in the study, yielding data on n = 152,767 patients, and representing 39.7% of the regional population. Higher than national average estimates were found for the age-adjusted prevalence of chronic diseases such as obesity/overweight (65.9% vs 63.4%), hypertension (11.9% vs 10.4%) and anxiety disorders (5.0% vs 3.8%), but a lower than national average age-adjusted prevalence of asthma (8.0% vs 10.2%) was also identified.

**Conclusions:**

This proof-of-concept study has demonstrated that the scope of data collected during patient visits to their general practitioners (GPs), facilitated through the Medicare-funded primary health care system in Australia, provides an opportunity for monitoring of chronic disease prevalence and its associated risk factors at the local level. Selection of sentinel sites that are representative of the population being served will facilitate an accurate and region-specific system for the purpose of population health planning at the primary care level.

## Background

Valid data on morbidity, at the regional level, is essential for planning of primary healthcare services that are specifically tailored to the needs, demands and requirements of the local population. The currently available peer reviewed literature indicates multiple avenues of population health surveillance including national surveys, administrative data and electronic health records. National surveys are surveillance methods that have commonly been used [1,2], but that demonstrate multiple shortcomings. Prevalence estimates obtained from national and state level surveys are based on self-reported measures, provided by respondents of these surveys, the accuracy of which is questionable [2]. Secondly, surveys do not include all aspects of health, and therefore do not provide a full and accurate picture of health [3], resulting in a lack of generalisability to smaller regional populations [4]. Additionally, different subgroups of the population may demonstrate different response rates to surveys, which impacts on the generalisability of the survey data to the general population [5,6]. Surveys, however, provide prevalence rates comparable to routinely collected clinical administrative data [1-3], indicating that clinical administrative data is a potential avenue for surveillance. The use of information obtained from administrative data, including primary care medical records (data collected during general practitioner (GP) visits), physician billing, specialist visits, pharmacy data (prescription dispensation) and hospital data (inpatient/outpatient information); has been shown to provide reliable and valid prevalence estimates of chronic disease conditions [1-3,7,8].

Clinical administrative data is widely available [1] and its collection and reporting systems are currently in place in both primary care and tertiary levels of care [9]. Further, administrative data is validated by the clinical judgment of medical practitioners and may be generalisable to smaller/regional populations [10]. Patient data that is entered into electronic medical recording software at the point of contact with primary health care practitioners is often supported by diagnostic testing and clinical examination, and is thus likely to be more valid than self-reported health information [10]. The peer-reviewed literature hence provides a vast amount of current evidence on the effectiveness of utilising administrative and/or primary care data for population health surveillance; however, there exists a current lack of such data based disease monitoring models in Australia.

The Sentinel Practices Data Sourcing (SPDS) project aimed to implement a sentinel site surveillance system within the Illawarra-Shoalhaven region of the state of New South Wales (NSW) in Australia to obtain a region-specific prevalence of chronic diseases and mental health disorders through the use of patient data obtained during primary care patient interactions. A pre-tested method of data extraction [10] was used, aimed at informing the population health planning within health service catchments of regional Australia.

## Methods

**Figure 1:**
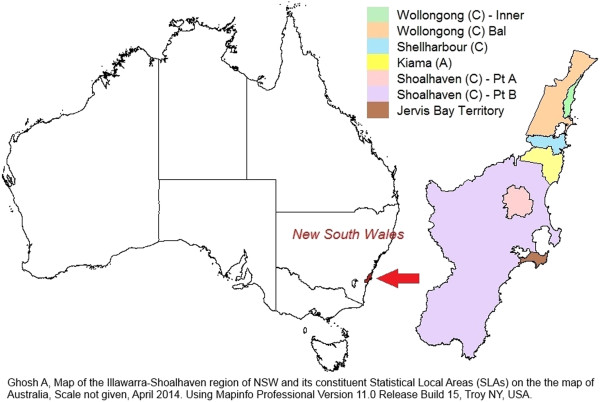
Illawarra-Shoalhaven region of NSW and its Statistical Local Areas (SLAs) on the map of Australia.

**Figure 2:**
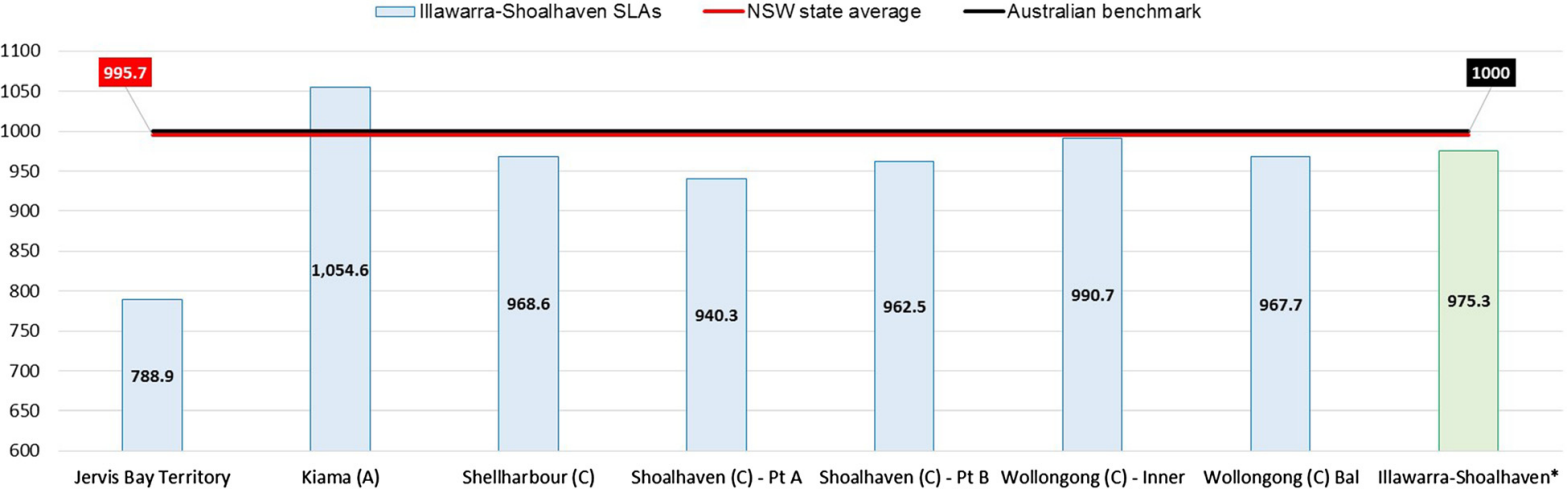
**Socioeconomic snapshot of the Illawarra-Shoalhaven region: Index of Relative Socio-Economic Disadvantage (IRSD) scores compared with NSW state and Australian national averages.** *Illawarra-Shoalhaven score is a population weighted average of individual SLA scores.

The study was conducted in the Illawarra-Shoalhaven region of the state of NSW in Australia (Figure 1). Within NSW there are 15 Local Health Districts which are responsible for the acute, sub-acute and tertiary care service delivery in the state through the public hospital system; and 17 Medicare Locals which are responsible for the primary healthcare planning and delivery for their constituent regions [10]. Unlike other regions, the geographical catchment boundary of the Illawarra-Shoalhaven Local Health District (ISLHD) is the same as that covered by a single Medicare Local namely the Illawarra-Shoalhaven Medicare Local (ISML); which places the region in a unique and advantageous position in terms of planning and implementing a chronic disease surveillance system. Additionally the region has a diverse socio-economic profile and has pockets of both higher and lower socio-economic disadvantage, comparing the Index of Relative Socio-Economic Disadvantage (IRSD) scores between the region and for Australia as a whole (Figure 2). IRSD is a composite summary measure constructed by the Australian Bureau of Statistics (ABS) for all regions in Australia and is a based on income, educational attainment, employment status, occupation type, family structure, dwellings, house ownership, marital status and ethnicity [11].

The study undertook secondary analysis of administrative data through extraction of de-identified clinical patient information and the project was rolled out in 4 phases: -

### Phase 1: Practice recruitment

The study aimed to recruit 12–18 practices within the Illawarra-Shoalhaven region based on the Statistical Local Area (SLA) geography and the demographic profile of the catchment. Eligible practices were identified by requesting the Illawarra-Shoalhaven Medicare Local (ISML) for a list of the region’s practices that fulfilled the following inclusion criteria (n = 52):

✓ location in one of the 7 SLAs that represent the Illawarra-Shoalhaven region;

✓ multiple (more than 1) GPs working at the practice site (solo practitioner sites are likely to have smaller patient numbers);

✓ employment of either more than one full-time GP or more than two part-time GPs (i.e. who work for at least 20 hours a week);

✓ additional criteria: -

• installation of a clinical auditing software package on desktop software or a desire to install and use the Pen Computer Systems (PCS) Clinical Audit Tool: ™(PCS CAT) (multiple licensing for PCS CAT has been procured by ISML and is therefore freely available to all general practices within its catchment); and

• a willingness to provide de-identified practice data extracts to the researchers for surveillance purposes.

Seventeen general practices in the catchment volunteered and consented to participate in the study (response rate = 33%). Only two electronic medical record software packages were being used by participating practices in the study, either Medical Director™ (n = 8) or Best Practice™ (n = 9).

### Phase 2: Data cleansing and enhancement of data accuracy

Recruited practices undertook comprehensive “Data Cleansing” training to understand the usage and the various functionalities of PCS CAT, and to update and clean the data stored in their clinical systems. With the consent of the primary GPs and managers within each practice, the “Data Cleansing” training was conducted by the researchers. The cleaning of practice records improves searches (in both the practice electronic medical record software program and the PCS CAT), to identify patients with particular conditions and thus to target health research and patient management activities. This data cleaning process allowed the complete patient database that had been entered during GP consultations to be identified when searching for specific variables. The data cleansing phase of the study was conducted using the data maintenance utility tools which are available within both the GP electronic medical record software programs used in the study. Data cleansing included: -

• encouraging all practice staff to use the ‘drop down box functionality’ of their clinical software to define and code all medical diagnoses and other sections of the patient record;

• strictly avoiding free text entries in all sections of the patient record;

• finding all identifiable free text non-coded past medical history items, and either linking them to appropriate coded items or replacing them with the correct coded item; and

• coding all inactive patients as ‘Inactive’ (an ‘active patient’ is one who has attended the practice three or more times in the past two years as defined in the Royal Australian College of General Practice: Standards for general practices [12]).

### Phase 3: Data collection

Patient data that had been de-identified by practice employees was extracted to a database. Data items extracted from general practice clinical systems included: demography (population by age and sex and population geography including postcodes and suburbs), chronic disease surveillance items (hypertension, type 2 diabetes mellitus, depression, anxiety, COPD, asthma, congestive heart disease, stroke, osteoarthritis, osteoporosis, high Body Mass Index (BMI) – overweight and obese), and Medicare Benefits Schedule (MBS) items uptake relevant to primary care services for GP and other non-referred attendances. A cleaned, de-identified PCS CAT data extract was performed in September 2013 for all recruited practices which included all information obtained from patient interactions in the preceding 24 months for all diagnosed pathologies, clinical variables such as anthropometric measures, and patient demographic information such as age, sex, geographical location of residence (postcodes and suburbs) and indigenous status.

### Phase 4: Data collation and analysis

The research team collated all extracted data, cross-matched residential suburb and postcode information with health and clinical information using de-identified unique link ID tags, converted all resultant information into usable database formats, and then analysed the datasets using Microsoft Excel (V2007: Microsoft Corporation, Redmond Washington, USA). The final datasets hence included clinical diagnosis and patient demographic information as entered by GPs within each participating practice. Basic epidemiological measures, including age-specific prevalence and total prevalence were calculated for all major disease conditions. The prevalence figures were compared against comparable indicators reported for the same age groups by the Australian Health Survey (AHS) 2011–12 conducted by the ABS [13].

The age-specific disease prevalence figures obtained from the study sample and the estimated national prevalence figures reported by the AHS 2011–12 were then age-standardised using the 2011 estimated resident population of Australia [14]. Comparisons across age-standardised prevalence were conducted for all major chronic conditions that the SPDS project is targeting for regular surveillance namely; obesity, overweight, diabetes mellitus, hypertension, asthma, mental health disorders such as clinically diagnosed depression and anxiety disorder, coronary heart disease, stroke, and chronic bone diseases such as osteoarthritis and osteoporosis. Both Microsoft Excel (V2007: Microsoft Corporation, Redmond Washington, USA) and the PCS CAT tool (v.3.1: pencs.com.au) were used for graphical illustration of demographic data and age-specific disease prevalence.

The study undertook secondary analysis of administrative data through extraction of de-identified clinical patient information. The study was performed with the approval of the Human Research Ethics Committee (Health and Medical) of the University of Wollongong (HE 12/447). Written informed consent was not obtained from individual patients due to the retrospective nature of the study design, however all data was exclusively extracted and de-identified by trained practice clinical staff only.

## Results

The number of patients that had visited the 17 general practices within the previous 24 months (September 2011 to September 2013) was 164,435 (152,767 from within the Illawarra-Shoalhaven and 11,668 from outside of the catchment). The Illawarra-Shoalhaven catchment sample of 152,767 included 70,103 men, 82,506 women and 158 without an identified gender.

**Figure 3:**
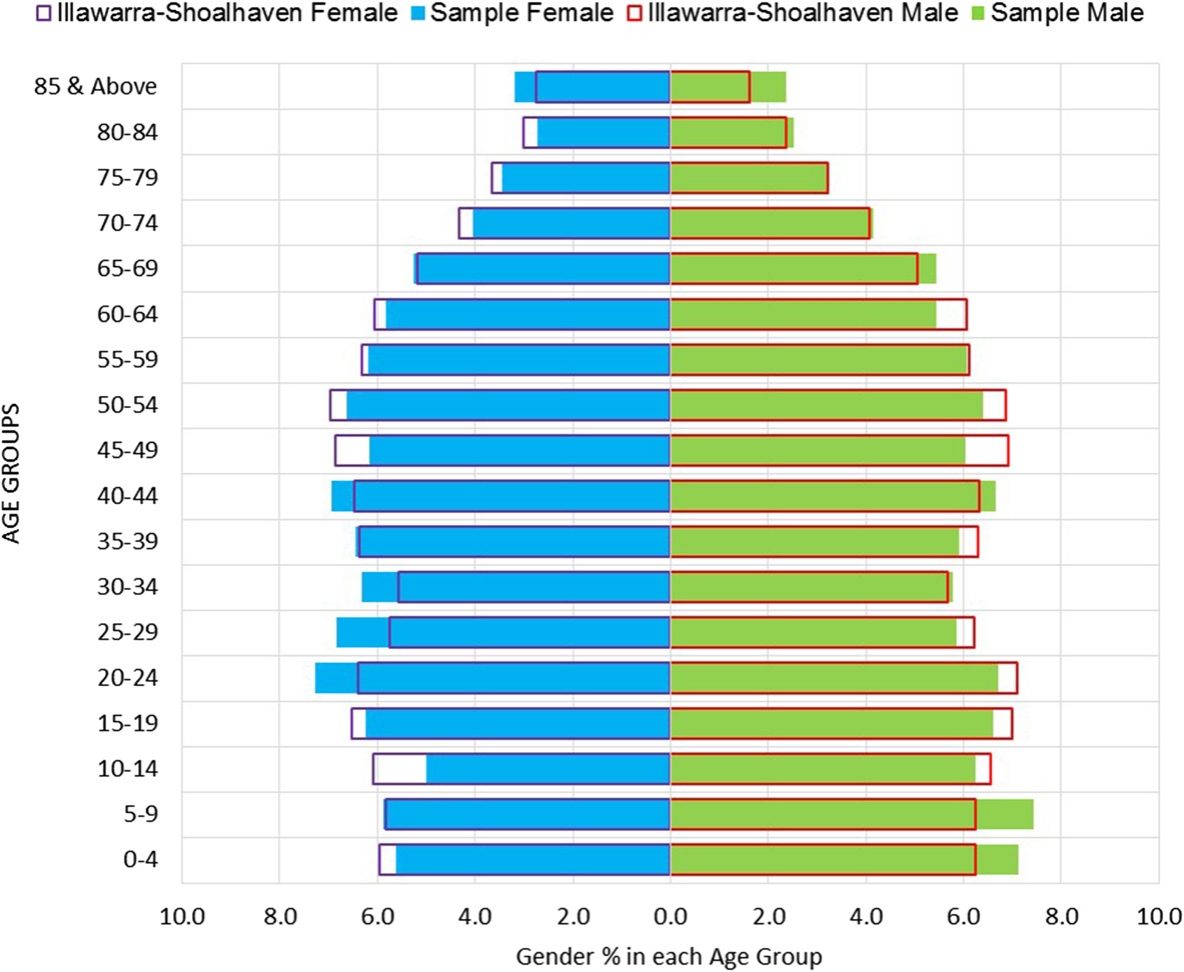
Population pyramid comparisons: study sample and the 2011 resident population of the Illawarra-Shoalhaven catchment.

While 144 patients did not have their age recorded, the median age for the study sample (n = 152,767) was 39 years (IQR = 20 – 58 years). Adults aged 20–24 years comprised the largest age group at 7% of the total sample, followed by the 40–44 year old age group (6.8%), and 5–9 year old children (6.6%). Older adults aged 65 years and above comprised 18.2% of the sample. The population pyramid of the study sample along with the comparison with the population structure of the 2011 estimated resident population of the Illawarra-Shoalhaven catchment is shown in Figure 3. The proportion of the local residential population of the SLAs that consulted the study practices during the study period is shown in Table 1[14]. The majority of the study sample (92.9%) were found to reside within the Illawarra-Shoalhaven catchment SLAs.

**Table 1:** Proportion of local population that had consulted the 17 general practices during the previous 24 months (September 2011 to September 2013)

**Illawarra-Shoalhaven SLAs**	**No. of patients from the SLA within the sample**	**Proportion of the sample from the SLA (%)**	Estimated Resident Population of the SLA, 2011*	**Proportion of the SLA population included in the sample (%)**
Jervis Bay Territory	6	0.0	387	1.6
Kiama (A)	19769	12.9	20832	94.9
Shellharbour (C)	44971	29.4	66054	68.1
Shoalhaven (C) - Pt A	15347	10.0	34444	44.6
Shoalhaven (C) - Pt B	26000	17.0	61599	42.2
Wollongong (C) - Inner	23351	15.3	104601	22.3
Wollongong (C) Bal	23323	15.3	96614	24.1
**Total**^**^**^	**152767**	**100**	**384531**	**39.7**

*ABS [14].

^^^Total number of patients in the data extract = 164,435; n = 152,767 after excluding patients residing outside the Illawarra-Shoalhaven SLAs and removing patients with geographic data inaccuracies.

**Figure 4:**
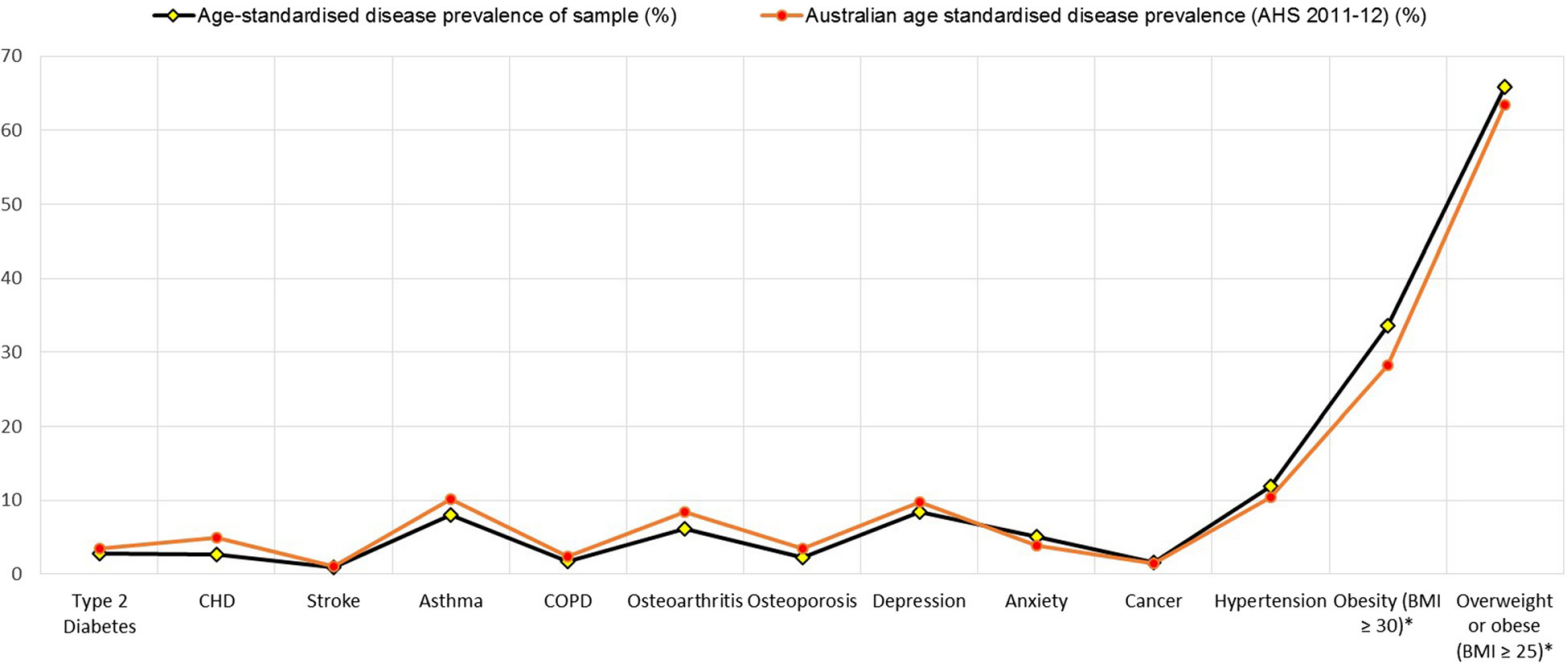
**Comparisons of age-standardised chronic disease prevalence between the study sample and Australian national averages.** *Includes adults only.

**Figure 5:**
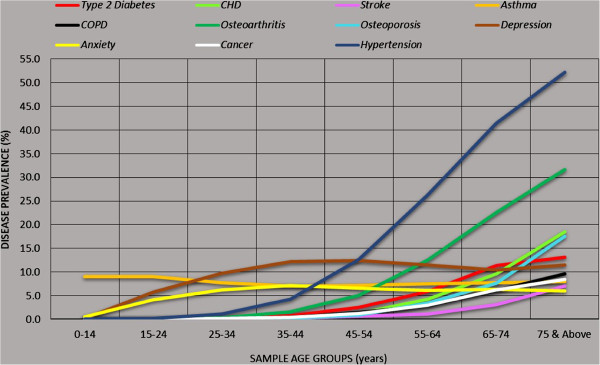
Age-specific prevalence of chronic diseases within the study sample.

The age-specific population and disease counts within the study sample for major chronic conditions and high BMI are shown in Tables 2 and 3 respectively, while the crude and age-standardised prevalence comparisons of the sample and the Australian national estimates [13] are shown in Tables 4 and 5 respectively. Overall the study sample population exhibits figures higher than Australian averages for the age-standardised prevalence of chronic conditions such as anxiety, cancer, hypertension, obesity and overweight/obesity (Figure 4). An illustration of age-specific burden of disease (Figure 5) indicates that prevalence (non-age standardised) of asthma and mental health conditions (depression and anxiety) are significantly higher amongst younger age groups compared to older adults.

**Table 2:** Age-specific population and chronic disease counts within the study sample during the last 24 months (September 2011 to September 2013)

**SLA**	**Age**	**Denominator (n)**	**Type 2 Diabetes**	**CHD**	**Stroke**	**Asthma**	**COPD**	**Osteoarthritis**	**Osteoporosis**	**Depression**	**Anxiety**	**Cancer**	**Hypertension**
Jervis Bay Territory	0-14	3	0	0	0	0	0	0	0	0	0	0	0
	15-24	0	0	0	0	0	0	0	0	0	0	0	0
	25-34	0	0	0	0	0	0	0	0	0	0	0	0
	35-44	2	0	0	0	0	0	0	0	0	0	0	0
	45-54	0	0	0	0	0	0	0	0	0	0	0	0
	55-64	1	0	1	0	0	0	0	0	0	0	0	0
	65-74	0	0	0	0	0	0	0	0	0	0	0	0
	75 & Above	0	0	0	0	0	0	0	0	0	0	0	0
	**Total**^**^**^	**6**	**0**	**1**	**0**	**0**	**0**	**0**	**0**	**0**	**0**	**0**	**0**
Kiama (A)	0-14	3348	0	0	0	262	0	0	0	6	20	1	0
	15-24	2572	1	0	1	190	0	0	2	118	57	1	5
	25-34	2138	0	0	2	137	1	4	1	115	80	3	13
	35-44	2450	12	5	6	147	4	30	3	191	94	8	115
	45-54	2558	39	18	15	143	13	96	19	218	104	24	286
	55-64	2751	124	100	26	180	29	267	80	227	113	59	691
	65-74	2094	203	152	53	134	72	355	145	152	71	128	844
	75 & Above	1844	209	284	126	137	132	437	329	147	63	122	980
	**Total**^**^**^	**19755**	**588**	**559**	**229**	**1330**	**251**	**1189**	**579**	**1174**	**602**	**346**	**2934**
Shellharbour (C)	0-14	9359	0	1	1	1028	1	1	0	31	64	2	5
	15-24	6826	0	0	1	598	1	7	2	483	334	3	19
	25-34	6381	10	4	4	543	8	39	4	714	478	5	55
	35-44	6191	33	15	13	530	33	131	6	816	511	16	245
	45-54	5378	106	64	32	490	85	336	34	767	434	26	627
	55-64	4340	168	158	50	456	161	629	126	598	323	68	1055
	65-74	3328	254	329	99	338	233	841	236	447	298	94	1276
	75 & Above	3156	302	638	215	343	405	1146	575	567	256	117	1504
	**Total**^**^**^	**44959**	**873**	**1209**	**415**	**4326**	**927**	**3130**	**983**	**4423**	**2698**	**331**	**4786**
Shoalhaven (C) - Pt A	0-14	2865	0	0	0	236	0	0	0	1	16	0	0
	15-24	2027	1	0	2	271	0	0	1	133	85	1	3
	25-34	1774	9	0	2	172	0	3	3	178	101	1	27
	35-44	1794	18	3	5	159	5	26	4	267	138	10	97
	45-54	1930	84	32	23	169	29	94	19	280	102	19	310
	55-64	1928	190	108	39	194	56	269	62	261	93	50	642
	65-74	1554	265	167	68	147	104	394	128	200	99	108	760
	75 & Above	1457	238	318	153	139	140	541	286	167	109	106	863
	**Total**^**^**^	**15329**	**805**	**628**	**292**	**1487**	**334**	**1327**	**503**	**1487**	**743**	**295**	**2702**
Shoalhaven (C) - Pt B	0-14	4344	0	0	0	307	3	0	2	5	15	0	2
	15-24	2970	0	0	0	302	0	0	0	129	86	2	4
	25-34	2486	3	1	0	188	2	5	3	249	94	10	39
	35-44	3032	26	7	5	186	1	44	5	366	163	18	119
	45-54	3486	78	45	20	201	29	178	27	410	173	72	429
	55-64	3767	222	134	28	191	81	430	110	403	168	171	921
	65-74	3297	403	323	86	221	149	736	200	299	152	291	1414
	75 & Above	2576	356	418	133	164	160	786	314	238	107	351	1292
	**Total**^**^**^	**25958**	**1088**	**928**	**272**	**1760**	**425**	**2179**	**661**	**2099**	**958**	**915**	**4220**
Wollongong (C) - Inner	0-14	3914	0	0	3	339	1	0	0	3	14	3	1
	15-24	2926	2	0	1	221	1	0	1	125	123	2	2
	25-34	3095	4	0	2	205	1	2	2	202	158	0	39
	35-44	3305	29	9	4	180	9	26	9	307	200	20	100
	45-54	3070	79	28	12	184	26	99	43	316	203	52	344
	55-64	2713	158	119	21	179	53	281	141	253	189	98	638
	65-74	2082	267	199	55	138	95	376	216	196	134	145	790
	75 & Above	2203	362	403	136	157	173	614	454	169	115	230	1186
	**Total**^**^**^	**23308**	**901**	**758**	**234**	**1603**	**359**	**1398**	**866**	**1571**	**1136**	**550**	**3100**
Wollongong (C) Bal	0-14	4411	0	0	1	370	9	2	0	6	24	0	0
	15-24	3141	1	1	3	270	17	4	0	193	167	2	11
	25-34	3121	6	0	6	236	13	7	5	379	269	4	33
	35-44	3080	23	13	7	208	35	47	7	461	311	12	174
	45-54	2850	96	49	18	186	86	162	28	402	263	40	440
	55-64	2489	199	143	43	161	137	380	105	319	209	78	780
	65-74	2036	236	216	89	134	229	538	177	210	156	129	888
	75 & Above	2180	290	420	189	127	284	729	403	254	150	207	1173
	**Total**^**^**^	**23308**	**851**	**842**	**356**	**1692**	**810**	**1869**	**725**	**2224**	**1549**	**472**	**3499**
Entire Sample	0-14	28244	0	1	5	2542	14	3	2	52	153	6	8
	15-24	20462	5	1	8	1852	19	11	6	1181	852	11	44
	25-34	18995	32	5	16	1481	25	60	18	1837	1180	23	206
	35-44	19854	141	52	40	1410	87	304	34	2408	1417	84	850
	45-54	19272	482	236	120	1373	268	965	170	2393	1279	233	2436
	55-64	17989	1061	763	207	1361	517	2256	624	2061	1095	524	4727
	65-74	14391	1628	1386	450	1112	882	3240	1102	1504	910	895	5972
	75 & Above	13416	1757	2481	952	1067	1294	4253	2361	1542	800	1133	6998
	**Total**^**^**^	**152623**	**5106**	**4925**	**1798**	**12198**	**3106**	**11092**	**4317**	**12978**	**7686**	**2909**	**21241**

^^^n = 152,767 but 144 patients without a recorded age were excluded, leaving n = 152,623 patients. All region stratified totals also exclude patients without a recorded age.

**Table 3:** Age-specific population and high BMI counts within the study sample during the last 24 months (September 2011 to September 2013)

**SLA~**	**Age**	Quantified BMI*: Denominator (n) [proportion of sample population (%)]	**Obese**	**Overweight/obese**
Kiama (A)	18-24	367 [19.7]	42	101
	25-34	366 [17.1]	75	178
	35-44	513 [20.9]	164	336
	45-54	782 [30.6]	283	557
	55-64	979 [35.6]	372	782
	65-74	958 [45.7]	354	726
	75 & Above	1125 [61]	275	735
	**Total**^**^**^	**5090 [32.4]**	**1565**	**3415**
Shellharbour (C)	18-24	510 [10.4]	132	257
	25-34	754 [11.8]	277	465
	35-44	778 [12.6]	366	602
	45-54	983 [18.3]	445	779
	55-64	832 [19.2]	367	689
	65-74	744 [22.4]	333	610
	75 & Above	775 [24.6]	228	524
	**Total**^**^**^	**5376 [16.0]**	**2148**	**3926**
Shoalhaven (C) - Pt A	18-24	528 [37.5]	90	187
	25-34	596 [33.6]	200	345
	35-44	696 [38.8]	277	507
	45-54	987 [51.1]	417	751
	55-64	1119 [58]	470	892
	65-74	993 [63.9]	417	798
	75 & Above	1004 [68.9]	261	676
	**Total**^**^**^	**5923 [50.0]**	**2132**	**4156**
Shoalhaven (C) - Pt B	18-24	468 [21.9]	55	135
	25-34	524 [21.1]	147	296
	35-44	823 [27.1]	252	541
	45-54	1258 [36.1]	414	889
	55-64	1492 [39.6]	517	1102
	65-74	1641 [49.8]	625	1290
	75 & Above	1481 [57.5]	376	990
	**Total**^**^**^	**7687 [37.0]**	**2386**	**5243**
Wollongong (C) - Inner	18-24	583 [26.1]	73	167
	25-34	729 [23.6]	161	355
	35-44	1048 [31.7]	295	681
	45-54	1179 [38.4]	414	846
	55-64	1042 [38.4]	372	782
	65-74	870 [41.8]	355	688
	75 & Above	1138 [51.7]	322	746
	**Total**^**^**^	**6589 [35.2]**	**1992**	**4265**
Wollongong (C) Bal	18-24	441 [19.1]	87	181
	25-34	634 [20.3]	252	422
	35-44	812 [26.4]	373	622
	45-54	1099 [38.6]	476	850
	55-64	970 [39]	467	804
	65-74	891 [43.8]	424	714
	75 & Above	1195 [54.8]	350	811
	**Total**^**^**^	**6042 [33.4]**	**2429**	**4404**
Entire sample	18-24	2897 [19.5]	479	1028
	25-34	3603 [19]	1112	2061
	35-44	4670 [23.5]	1727	3289
	45-54	6288 [32.6]	2449	4672
	55-64	6434 [35.8]	2565	5051
	65-74	6097 [42.4]	2508	4826
	75 & Above	6718 [50.1]	1812	4482
	**Total**^**^**^	**36707 [30.9]**	**12652**	**25409**

*Only includes patients with both height and weight recorded.

~ No patients identified in Jervis Bay Territory for this indicator.

^^^All region stratified totals also exclude patients without a recorded age.

**Table 4:** Crude prevalence proportions of chronic conditions in the study sample compared to Australian national averages

**Chronic Disease/Conditions (as defined and entered into electronic records by GP)**	**Crude prevalence in sample (%) (95% CI)**	**Australian crude disease prevalence (AHS 2011–12~) (%) (95% CI)**
Type 2 Diabetes	3.3 (3.26 - 3.44)	3.4 (3.09 - 3.71)
CHD	3.2 (3.14 - 3.32)	4.7 (4.39 - 5.01)
Stroke	**1.2 (1.12 - 1.23)**	1.1 (0.94 - 1.26)
Asthma	8.0 (7.86 - 8.13)	10.2 (9.58 - 10.82)
COPD	2.0 (1.96 - 2.11)	2.4 (2.09 - 2.71)
Osteoarthritis	7.3 (7.14 - 7.4)	8.3 (7.84 - 8.76)
Osteoporosis	2.8 (2.75 - 2.91)	3.3 (3.03 - 3.57)
Depression	8.5 (8.36 - 8.64)	9.7 (9.19 - 10.21)
Anxiety	**5.0 (4.93 - 5.15)**	3.8 (3.45 - 4.15)
Cancer	**1.9 (1.84 - 1.97)**	1.5 (1.29 - 1.71)
Hypertension	**13.9 (13.74 - 14.09)**	10.2 (9.72 - 10.68)
Obesity (BMI ≥ 30)*	**34.5 (33.98 - 34.95)**	28.3 (27.36 - 29.24)
Overweight or obese (BMI ≥ 25)*	**69.2 (68.75 - 69.69)**	63.4 (62.28 - 64.52)

*Adults Only.

~ABS [13].

Bold font indicates a higher prevalence than national estimates in the study sample.

**Table 5:** Age-standardised prevalence of chronic conditions in the study sample compared to Australian national averages

**Chronic Disease/Conditions (as defined and entered into electronic records by GP)**	**Age-standardised disease prevalence in sample (%)**	**Australian age-standardised disease prevalence (AHS 2011–12~) (%)**
Type 2 Diabetes	2.8	3.4
CHD	2.6	4.9
Stroke	0.9	1.1
Asthma	8.0	10.2
COPD	1.7	2.4
Osteoarthritis	6.1	8.4
Osteoporosis	2.2	3.4
Depression	8.4	9.7
Anxiety	**5.0**	3.8
Cancer	**1.6**	1.5
Hypertension	**11.9**	10.4
Obesity (BMI ≥ 30)*	**33.6**	28.3
Overweight or obese (BMI ≥ 25)*	**65.9**	63.4

*Adults Only.

~ABS [13].

Bold font indicates a higher prevalence than national estimates in the study sample.

## Discussion

In Australia, nationally representative data is available through the National Health Surveys (NHS) conducted by the Australian Bureau of Statistics (ABS) [15] and regionally through state surveys such as the annual New South Wales Population Health Survey [16]; however, extrapolations of these data to smaller geographical areas such as Local Government Areas (LGAs) and/or small area geographic regions within LGAs such as SLAs and suburbs is limited [10].

In 2011–12, the percentage of adults who saw a GP in the preceding 12 months varied across Australia ranging from 74% to 86%, with the Illawarra-Shoalhaven catchment recording the highest percentage nationally [17]. This indicates that extraction of patient and GP interactions over a 24 month period has the potential to include health information of almost the entire resident population of catchment regions such as the Illawarra-Shoalhaven; however, not all SLAs were evenly represented in terms of coverage in this study.

All general practice clinical and electronic medical record softwares utilise one of the several nationally validated health coding and medical classification systems such as SNOMED-CT, DOCLE, PYEFINCH and ICPC2+ [10]. These medical vocabularies enable recording of nationally/internationally recognised coded disease diagnosis, which also assists in maintaining accurate and consistent primary care clinical data that can be extracted and analysed [10]. Additionally, recent introduction of the Personally Controlled Electronic Health Records (PCEHR) in Australia, further requires general practices to “*work towards recording the majority of diagnoses for active patients electronically*” [18]. This enables accurate recording and easy identification of medical conditions and hence provides an opportunity for successful public health and chronic disease surveillance. However, Aizpuru et al. [19] suggest that chronic disease data from electronic health records provide a lower prevalence of conditions, as compared to health surveys because actions taken by physicians are often not recorded leading to cases being missed out. The limitations of primary care practice based data collection have also been illustrated in systematic reviews. Common problems include inconsistent diagnostic coding vocabulary of different clinical systems [20] and errors in data entry and recording [7,21]. Major barriers faced by general practice staff in this regard include difficulties with clinical coding of diagnoses; complexities with software applications; preference for entering free text rather than the pre-coded options; inadequate skills in information technology; time constraints; poor motivation and low prioritisation of data entry compared to other clinical duties; inconsistency of data entry; coding of a condition in order to justify choice of prescribed treatment; and the additional burden of including laboratory test results in patient records, as well as a need to enter a diagnosis, even in the early preclinical stages of the disease (Attard E, Ghosh A, Charlton K: Barriers faced by general practice staff in maintaining clean primary care databases: a systematic review, unpublished).

Studies conducted in Canada [22,23], Italy [24] and the UK [25] have demonstrated methods to improve the quality and accuracy of practice-based disease surveillance models. Keshavjee et al. [22] trained and employed ‘data managers’ in an attempt to standardise disease coding and de-identify patient information. Similarly, Griever et al. [23] employed a trained data entry clerk to check missing or incorrectly coded records. Cricelli et al. [24] trained GPs, themselves, in data entry and use of standard software. Pearson et al. [25] provided initial training and updates to all doctors and practice staff and carried out validation procedures such as verification of clinical coding, checking for rare diagnoses and those made outside the usual age and sex parameters through random validation visits to all participating practices.

A number of limitations to the study need consideration. The SPDS study identified various common data entry errors, including misspelt suburb names or postcodes that did not match the suburb entry, missing geographic information (postcodes and suburbs), missing values for age and sex, incorrect/mismatched entries within data entry fields such as height inserted in the weight field and/or vice-versa, and missing entries for weight and/or height measurements. While geospatial analysis of disease patterns is highly useful to target services towards areas of need [26], the SPDS data has highlighted difficulties in obtaining consistent information on patient residential postcodes and suburbs, including missing entries and mismatched entries, for example, a record with suburb of the Kiama (A) SLA and postcode of 3000 which is the incorrect postcode for this suburb. It was often unclear which variable to change in order to resolve this inconsistency and hence led to the deletion of such records from the analysis. Data quality and accuracy discrepancies required immense post-extraction data cleaning/editing efforts by the researchers which is vital to improve data linkage quality [27]. It is therefore imperative to undertake further research and technological innovation into improving utility and interface functionality of practice clinical desktop systems and creation of valid and easy to use advanced data aggregation systems which could vastly improve the processes of primary care clinical data extraction and modelling resulting in furthermore accurate prevalence estimation.

Both international literature and Australian evidence identifies a higher reported prevalence of overweight and/or obesity within primary care settings [28,29]. It has been argued that obese patients are more likely than healthy or underweight patients to visit their physician and also more likely to be weighed and measured by practice staff and clinicians. This results in lower population denominators for obesity and overweight, as also seen in our study (Table 3), and arguably higher prevalence figures. This is another limitation of the proposed method of surveillance.

The seventeen general practices recruited from the major SLAs within the Illawarra-Shoalhaven region include approximately 40% of the resident population of the catchment area but generalisability of the findings to the general population of the Illawarra-Shoalhaven region cannot be assumed. Additionally there was a clear coverage disparity between the 7 Illawarra-Shoalhaven SLAs with high representation of Kiama (A) and Shellharbour (C) residents, moderate representation of the Shoalhaven (C) - Pt A and Shoalhaven (C) - Pt B residents and low proportional share of Wollongong (C) Inner and Wollongong (C) Bal SLAs within the study sample (Table 1). This can be attributed to the recruitment of practices that voluntarily consented to participate rather than routine surveillance as such. Thus, disease prevalence estimates drawn from the study sample may not be representative of the true population disease status for the region.

Another limitation to the study is that it only investigated the interaction between one extraction tool (the PCS CAT) and two general practice electronic medical record (EMR) software systems (Best Practice™ and Medical Director™). Although these are the most commonly used systems in Australia, the findings cannot be extrapolated to other systems. Additionally the validity of a PCS CAT extract has not been completely investigated. While the tool is co-developed by the Royal Australian College of General Practice (RACGP), the peak body of general practice in Australia and is advocated by them as an integrated product aimed at improving the way patient information can be used to better inform decisions in both clinical and business settings [30]; to date there has not been any empirical validation of the PCS CAT as a general practice data extraction tool. Further research into validation of the PCS CAT extract and the assessment of its agreement with manual data review/audit is required. A final limitation is that we only included data that could potentially be extracted from the electronic medical record software programs. While the data cleansing phase of the study focused heavily on avoidance of any free text entered into medical or clinical notes by GPs and practice staff; if a practitioner still made free text entries rather than using the codable sections of the record, then neither the extraction tool nor our manual case record reviews/audits would be able to detect those cases.

Despite these limitations the SPDS study has significant implications for public health planning, primary health care delivery and epidemiological research. Apart from ongoing chronic disease surveillance, the study methodology and protocol also has the potential to provide evidence-based direction to population health planning strategies aimed at addressing the local health needs of regional areas of Australia. The most recently reported planning documents for the Illawarra-Shoalhaven region of NSW, both from the Local Health District level [31] and the Medicare Local level [32], illustrate disease rates and health status indicators drawn from statistically modelled estimates from the 2006–07 Australian National Health Surveys. These figures are significantly outdated for planning purposes in 2014 and their generalisability for regional and smaller area disease prevalence and health status is questionable [10]. The proposed surveillance system also provides opportunity for monitoring trends in chronic disease prevalence across regular time intervals and promotes the engagement of general practice staff and clinicians in maintaining primary care clinical data quality and accuracy. The inclusion of a larger number of sentinel sites that are generalisable to the population being served would provide an accurate and region-specific system for the purposes of population health planning at the primary care level in order to improve the overall health of the community.

## Conclusion

This study has demonstrated that extraction of patient clinical data from general practice settings is both a feasible and valid method to obtain a region-specific estimate of the prevalence of chronic diseases and mental health disorders within regional NSW, Australia. General practices that agreed to participate were included in the study, however further sampling methodology is required to identify which sentinel sites would provide an accurate and truly representative surveillance system. Technological updates/changes to general practice clinical software systems are recommended to improve functionality and data quality within general practice databases. Drop down menus with fixed nationally recognised lists of suburb names, cross matched with correct geographical concordance postcode and state information is currently lacking within the general practice clinical software systems. Additionally, making age, sex, postcode and suburb mandatory fields for creating a new patient record could eliminate the issue of missing data for these essential socio-demographic variables. Investment in computer skills and data entry training for general practice staff and advancements in data aggregation instruments are essential to improve quality of clinical data and their collection methods for effective utilisation by researchers and population health planners for surveillance purposes. Annually obtained chronic disease prevalence figures through the surveillance methodology implemented by the SPDS project, could provide more updated and granular health information for prompt health service planning.

## Abbreviations

GPs: General practitioners; ISML: Illawarra-Shoalhaven Medicare local; ABS: Australian Bureau of Statistics; LGAs: Local Government Areas; SLAs: Statistical Local Areas; MLs: Medicare Locals; NHS: National Health Surveys; ISLHD: Illawarra Shoalhaven Local Health District; PHIDU: Public Health Information Development Unit; NSW: New South Wales; PCS CAT: Pen Computer Systems Clinical Audit Tool; SNOMED-CT: Systematized Nomenclature of Medicine Clinical Term; DOCLE: Doctor Command Language; ICPC2+: International Classification for Primary Care 2+; AHS: Australian Health Survey; MBS: Medicare Benefits Schedule.

## Competing interests

The author(s) declare that they have no competing interests.

## Authors’ contributions

AG formulated the study design and conceptualisation, performed data extraction and all statistical analysis, conducted data interpretation, carried out literature search and collated contributions from co-authors to draft the paper. AG also carried out all manuscript revisions as per peer-reviewer’s comments. KEC contributed to study design and conceptualisation, provided editorial input, conducted data interpretation and carried out literature search. LG trained and educated practice staff in undertaking the data cleansing of their practice clinical database and carried out literature search. MB reviewed the statistical methodology and data analysis, and contributed to editing the final paper manuscript. All authors read and approved the final manuscript.

## Pre-publication history

The pre-publication history for this paper can be accessed here:

http://www.biomedcentral.com/1471-2458/14/557/prepub
